# The effect of whisker movement on radial distance estimation: a case study in comparative robotics

**DOI:** 10.3389/fnbot.2012.00012

**Published:** 2013-01-02

**Authors:** Mathew H. Evans, Charles W. Fox, Nathan F. Lepora, Martin J. Pearson, J. Charles Sullivan, Tony J. Prescott

**Affiliations:** ^1^Department of Psychology, Sheffield Centre for Robotics, University of SheffieldSheffield, UK; ^2^Bristol Robotics Laboratory, University of the West of EnglandBristol, UK

**Keywords:** active sensing, touch, whisker, robot, biomimetic, comparative, perception, classification

## Abstract

Whisker movement has been shown to be under active control in certain specialist animals such as rats and mice. Though this whisker movement is well characterized, the role and effect of this movement on subsequent sensing is poorly understood. One method for investigating this phenomena is to generate artificial whisker deflections with robotic hardware under different movement conditions. A limitation of this approach is that assumptions must be made in the design of any artificial whisker actuators, which will impose certain restrictions on the whisker-object interaction. In this paper we present three robotic whisker platforms, each with different mechanical whisker properties and actuation mechanisms. A feature-based classifier is used to simultaneously discriminate radial distance to contact and contact speed for the first time. We show that whisker-object contact speed predictably affects deflection magnitudes, invariant of whisker material or whisker movement trajectory. We propose that rodent whisker control allows the animal to improve sensing accuracy by regulating contact speed induced touch-to-touch variability.

## 1. Introduction

Many robots have been developed for understanding whisker sensing (Prescott et al., 2009; Evans et al., 2012). Though each has expanded our understanding in certain ways, it is difficult to apply the results in a general way to other paradigms or wider applications. Choices made in the development of robotic hardware specify the kind of questions that can be answered by that platform. For example, robots with high degrees of freedom allow research into the effects of whisker movement on sensing, but this movement is not precise enough to expose the underlying mechanisms of whisker sensing.

A complementary robotics approach, similar to approaches used in the biological sciences (Kappers et al., 1936; Kardong, 2006), allows the development of robots where the results from one platform can inform the experiments on another. Great progress can be made, both by performing experiments on appropriate platforms and ensuring that results inform general conclusions. Results from robotics in turn may provide insights for neuroscience. A key example where such an approach may be fruitful is in understanding the effect whisker movement has on sensing.

In this paper we will briefly introduce rodent whisker movement control, and whiskered robots that model these systems will be reviewed. A comparative robotics approach is described, outlining a path for addressing some of the questions from biology in a more explicit and effective manner. Specifically, what is the effect of whisker movement on radial distance estimation?

### 1.1. Active whisker touch sensing in rodents

Whiskers are found in almost all terrestrial mammals, *Homo Sapiens* excepted, and some marine mammals (Ahl, 1986). Although whiskers are hairs, their structure is highly specialized, with regards to their surface structure and mechanical properties, in transferring contact information to the hair follicle for tactile sensing (Chernova and Kulikov, 2011). For example, whiskers vary in length, thickness, shape, and stiffness between species depending on animal size or how the whiskers are used (Sarko et al., 2011).

Rats typically have around 30 prominent whiskers on each cheek (or mystacial pad), arranged in a regular grid of rows and columns (Ahl, 1986). These large *macro*-vibrissae vary in length and width across the whisker pad, from the largest [2–40 mm in length (Diamond et al., 2008)] in the most caudal column down to the smallest in the rostral column. A dense array of 40–70 smaller *micro*-vibrissae (a few mm in length) are located around the lips (Brecht et al., 1997). Physical differences between the whiskers affect their mechanical properties, such as their bending and damping characteristics (Hartmann et al., 2003), which could have repercussions for sensing, a critical consideration when building artificial whiskers.

Whiskers can only encode information about objects when they make contact with them. To gather information about the world, rodents sweep their whiskers through the air, and bring them on to surfaces in the environment. This back and forth sweeping movement of the whiskers [called “whisking” (Welker, 1964)] has been the subject of a great deal of research. A single “whisk” is defined as one cycle of whisker protraction (forward movement) and retraction (backward movement), and without perturbation rats typically whisk in short bouts of ≈10 cycles, at around 5–8 Hz (Carvell and Simons, 1990).

Though initially thought to be very regular (Semba and Komisaruk, 1984), recent studies using optoelectronic monitoring techniques (Bermejo et al., 2002) and high speed videography (Sachdev et al., 2002; Towal and Hartmann, 2006) has revealed that rat whisking can be highly irregular and complex. It is full of asynchronies, where different whiskers are protracted by different amounts (Sachdev et al., 2002) and asymmetries, where the whiskers on either side of the head are moved out of phase with one another (Towal and Hartmann, 2006). These irregular movements are thought to be the result of active sensing strategies (Hartmann, 2001; Berg and Kleinfeld, 2003; Mitchinson et al., 2007).

Among other parameters (Towal and Hartmann, 2008), rats control the spread and contact force of whiskers to ensure even, light contacts across the whisker array. Specifically, rats seem to use particular strategies for sensing, such as the rapid cessation of protraction upon initial contact with a surface, and contact induced asymmetry in the whisker movements, where a whisker contact on one side of the rat's head causes an increase in the protraction of whiskers on the side contra-lateral to contact (Grant et al., 2009). Together these efforts are grouped into a strategy described as minimal impingement (hereafter MI), maximal contact (Mitchinson et al., 2007).

In addition, head movement greatly effects the velocity of whisker contacts (Grant et al., 2009), and whisker movement is controlled to sweep space in anticipation of head movement (Towal and Hartmann, 2006). Though some have been identified (Grant et al., 2009), it remains unclear which components of whisker movement are actively controlled by the rat, which are artefacts arising from limitations of biological systems, and which if any are important for sensing. For example, do rats change their whisking frequency to improve the discrimination of particular surfaces, or because their muscles cannot maintain high frequencies of whisking for prolonged periods of time?

### 1.2. Active whisker touch sensing in robots

A number of software and hardware models have been developed to better understand whisker sensing. There are many reasons why modeling a system is an important step toward understanding, and why synthetic models (models built in software or hardware) in particular are so useful (Rosenblueth and Wiener, 1945; Mitchinson et al., 2010). For example, in a model whisker movement can be precisely controlled to determine the effects any changes have on whisker deflections and subsequent analysis.

Whiskers have been modeled simply as elastic beams (Salisbury, 1984; Young et al., 2003). Though progress has been made very recently in more precise computational modeling of whiskers (Quist and Hartmann, 2012), their small size make accurate simulation difficult. A more straightforward method is to build artificial whiskers and mount them on robots. Whiskered robots have been broadly reviewed recently in Prescott et al. (2009). Specifically focusing on whisker actuation mechanisms, and the effect these have on sensing, early models were static and provided binary contact vs. no contact reports (Schiebel et al., 1986; Jung and Zelinsky, 1996). Hinged whiskers were used to infer the location of tip contact through potentiometer readings (Russell, 1992). Emulating earlier modeling work, elastic beam equations have been used by a number of researchers to infer the location of contact along an artificial whisker, and in turn the curvature of a surface with whiskers mounted on robots (Russell and Wijaya, 2003, 2005), rotational DC motors (Kim and Moller, 2004, 2007), or a set screw (Solomon and Hartmann, 2006). In a unique design Wilson and Chen (1995) used a pair of pressurized tubes laid end to end as a whisking mechanism, and a closed loop control system to infer whisker tip contact location in space.

In more biomimetic (Vincent et al., 2006) robots (such as in, Seth et al., 2004; Fend et al., 2005; Meyer et al., 2005; Pearson et al., 2007; Lepora et al., 2012b) multiple degrees of freedom are included as whiskers are often actuated, as well as being mounted on mobile robot platforms. This increased whisker movement makes texture discrimination difficult (Fend et al., 2003), especially in conditions where whisker motion varies from trial to trial (Fox et al., 2009). To address this point further more complex whiskered robots, with individually actuated whiskers have been developed in recent years for investigating biomimetic whisker control strategies (such as MI discussed earlier, Pearson et al., 2011), and how these strategies may improve texture discrimination (Lepora et al., 2010b; Sullivan et al., 2012).

### 1.3. Whisker materials

The material a whisker is made from has a critical influence on the way a whisker interacts with a surface, and as a result the nature of the deflections created at the whisker base (Hartmann et al., 2003). Whiskers are specialized sensory elements for aiding tactile sensing in the hair follicle, differing in structure from other mammalian hairs to ensure strength and stiffness (Chernova and Kulikov, 2011; Sarko et al., 2011). Rat whiskers have evolved to have excellent mechanical properties for transferring tactile information to the follicle during sensory exploration (Chernova and Kulikov, 2011). Specifically, rat whiskers are stiff when moved in air but bend in contact and are highly damped with damping ratios ζ of 0.11:0.19 and Young's modulus *E* of ≈3–4 GPa (Hartmann et al., 2003). This ensures that the whiskers do not oscillate when whisked in air, which can add noise to the deflection signal and make contacts difficult to detect. This damping also increases when the whisker is in the animal, as observed in that contact induced oscillations are smaller in whisking rats than isolated whiskers (Hartmann et al., 2003). Whiskers are tapered, which has certain advantages [some are described in detail by Williams and Kramer (2010)], so it is important that artificial whiskers taper if they are to appropriately mimic the biological system. In artificial systems whisker material and morphology have also been shown to be important for texture discrimination (Lungarella et al., 2002; Fend et al., 2006).

### 1.4. Radial distance to contact estimation

Estimating the radial distance to contact (in this paper, from the base of the whisker) allows an agent to determine whether an object has made contact with a whisker at the tip or the shaft, which is important for texture discrimination, and to discriminate between contacts with the surfaces or corners of objects. Measuring the location in space of multiple contacts over time allows an agent to reconstruct the contours of an object or perimeters of the environment (Fox et al., 2012). Radial distance estimation has been demonstrated in rodents (Szwed et al., 2006), and approached by many researchers. Theoretically, radial distance to contact estimation along a beam is a solved problem (Solomon and Hartmann, 2006, 2011), as long as whisker movement is precisely controlled and the physical properties such as size, taper, and elasticity are known. In an applied robotic setting these parameters are not always known precisely, therefore a more data-driven approach is appropriate (Evans et al., 2010a; Lepora et al., 2010a). In this paper we use a feature-based radial distance estimation method, essentially extracting analogous information to the bending moment at peak protraction, but using regression to determine the relationship between this value and radial distance to contact.

Feature-based classification involves finding invariant features in the data that correspond to parameters in the real world. For example, using scale invariant feature transformation (SIFT) algorithms in vision (Lowe, 1999; Juan and Gwun, 2010). Feature extraction has also been demonstrated in biological sensing systems. Frog prey capture is based on the principle of feature detection, with responses elicited for any object matching the size and angular velocity of a fly (Lettvin et al., 1959). In the rat whisker system some researchers have reported cells that respond to “kinetic features” in whisker deflections (Petersen et al., 2008). An advantage of this approach is that it reveals how different whisker movement patterns affect the extracted features, and may allow the measurement of numerous features to classify a range of whisker-object contact parameters simultaneously in future.

### 1.5. A comparative robotics approach

As robots become more complex, they become more difficult to control. As this progression continues it may be difficult to conduct experiments that address fundamental questions about whisker-object interactions. In this paper we present a complementary robotics approach. Here, a group of different robots are used to address the same problem of radial distance to contact estimation, allowing a direct comparison of whisker materials and actuation methods. This approach may help in understanding radial distance to contact estimation more generally, invariant of whisker material or actuation method.

Three robots were used for comparison. Firstly, an XY positioning robot moves objects onto an artificial whisker sensor in an accurate and highly repeatable manner, allowing the collection of large amounts of whisker deflection data. This approach provides the opportunity for a better understanding of the nature of whisker-object contacts (expanding on previous preliminary work in Evans et al., 2010a,b). Data collected on the XY positioning robot is used to systematically train and test a classifier under a range of contact conditions, and extract features for radial distance to contact and contact speed estimation. Secondly, SCRATCHbot (an acronym of Spatial Cognition and Representation through Active TouCH, Pearson et al., 2010) is a mobile whiskered robot which approximates the degrees of movement of an exploring rat. Actuated whiskers are mounted on an articulated “neck,” which is in turn fixed to a mobile base. SCRATCHbot is used here in a “head-fixed” protocol to show how the classifier and features developed on the XY positioning robot can be applied to data from a less restricted whisking robot. Thirdly, CrunchBot is a mobile whiskered robot with stationary whiskers (Fox et al., 2011). This robot has fewer degrees of freedom than SCRATCHbot, allowing more straightforward robot control and data collection in a mobile setting. Testing the feature-based classifier on CrunchBot evaluates whether this approach can be robustly implemented on a mobile robot. In addition to separate actuation methods, all three robot platforms utilize different whisker materials. This change allows for the comparison of whisker materials in the same radial distance estimation task, and tests the classifier's robustness to this change. A great deal of effort was made to find materials for artificial whiskers that would match the mechanical properties of real whiskers at different scales, such as stiffness and bending characteristics. Additional concerns are toughness and the ability to appropriately shape the whisker.

## 2. Materials and methods

### 2.1. Robot platforms

#### 2.1.1. XY positioning robot platform

**Figure 1:**
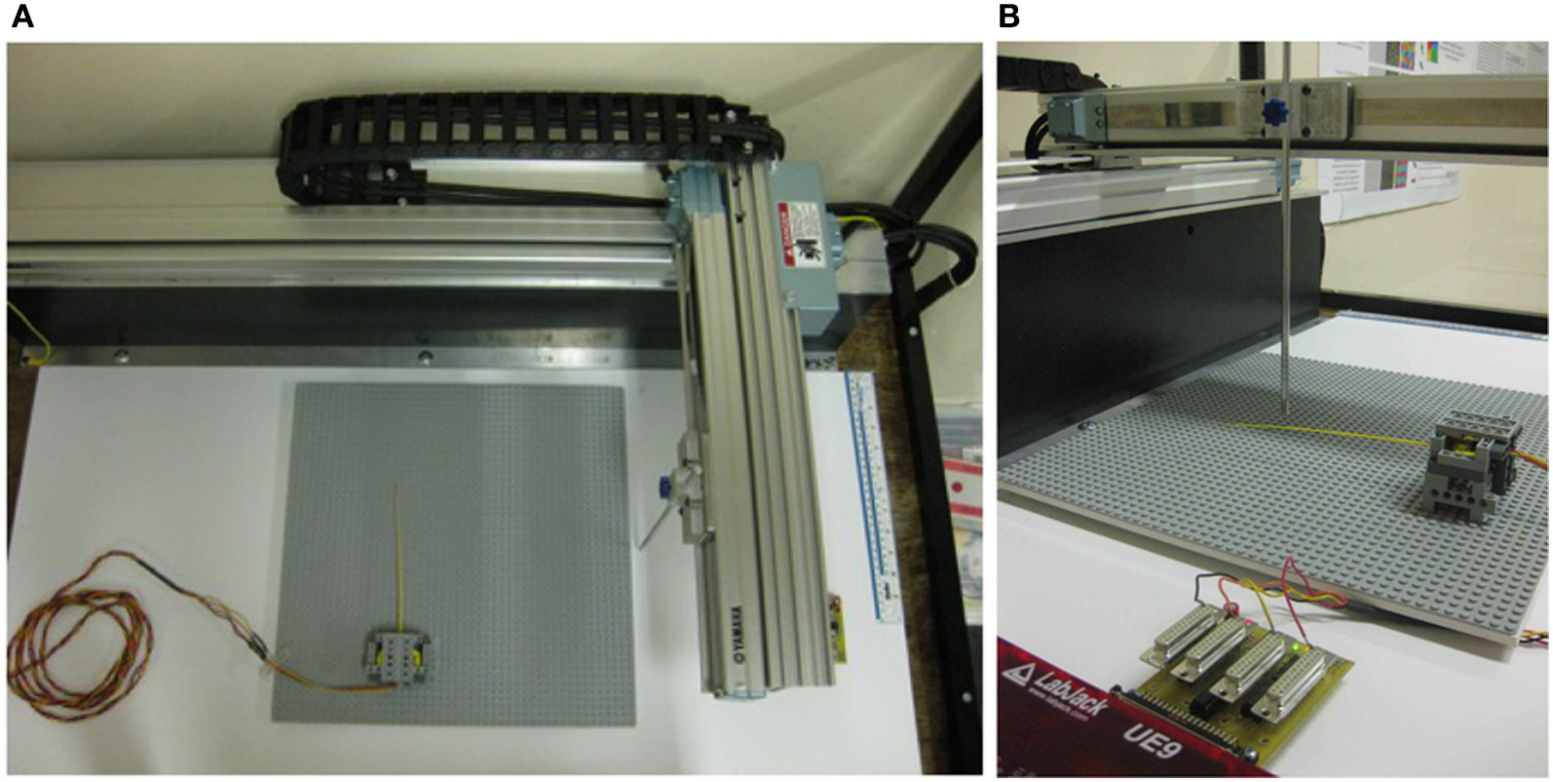
**The XY positioning robot. (A)** From above, to show the range of movement available. **(B)** From the side. A narrow, rigid aluminium bar was moved into the whisker perpendicularly, from a clockwise or anticlockwise direction.

A Cartesian robot (see Figure 1) was chosen as it is capable of a wide range of movement, is very accurate and can move at speeds which approximate scaled rat-whisk velocities. Deflections for the whisker are streamed to a PC, and can be processed in real time to control subsequent movement of the positioning robot. The robot (Yamaha-PXYX, Yamaha Robotics) has a movement range of 350 × 650 mm, and can move up to 720 mm/s. Repeatability of the robot is ±0.01 mm, and the maximum load it can carry is 1.5 kg. Objects are carried by the robot into an artificial whisker fixed to the table, as this allows us to control the contact as carefully as possible. Moving the whisker into an object would subject the sensor to trajectory-dependent accelerations that would cause more complex effects such as whisker oscillations. Subsequent robots described in this paper allow for exploring these trial to trail variations and their effect on sensing. A controller (Yamaha RCX 222, 2-axis robot controller) takes instructions from a PC through an RS232 cable, and the controller interprets the instructions, completes path integration, and drives the motors. Instructions for the robot are generated inside a MATLAB (mathworks.com) loop, and can be easily updated during robot operation, depending on the whisker input.

#### 2.1.2. SCRATCHbot robot platform

**Figure 2:**
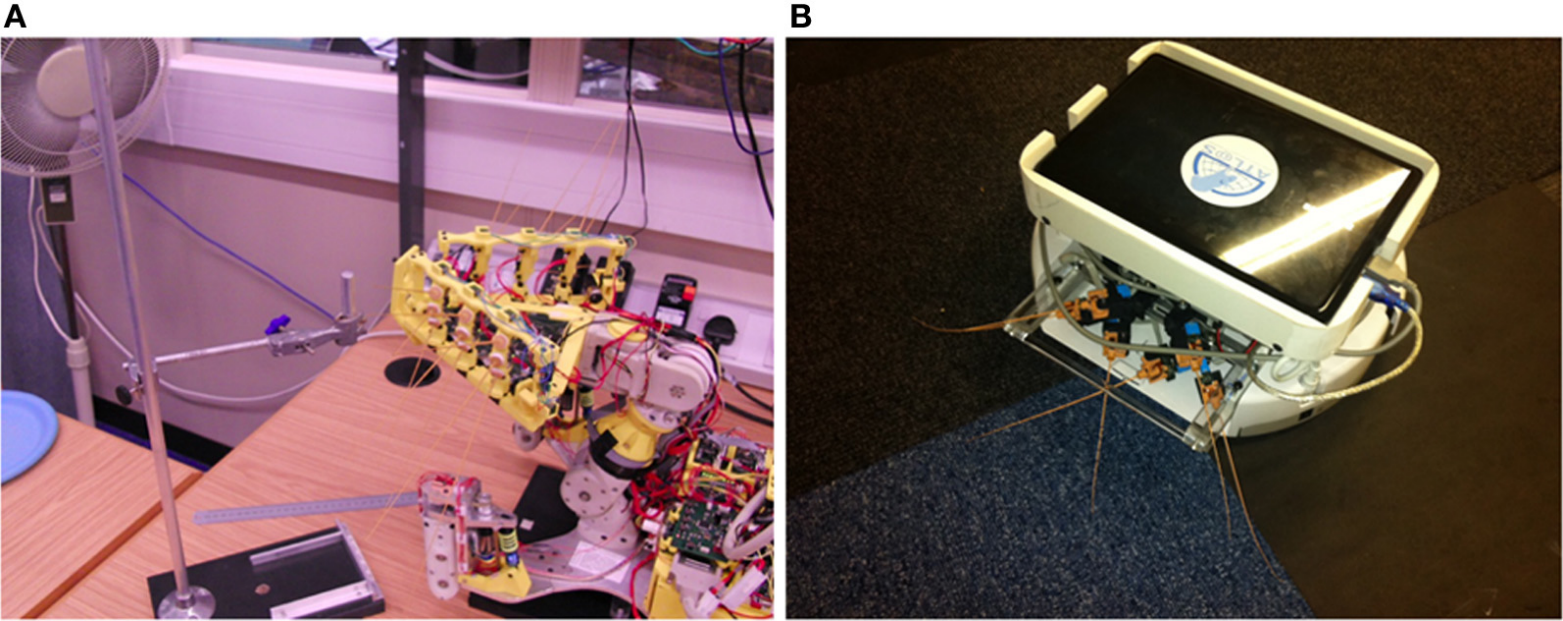
**(A)** The SCRATCHbot whiskered mobile robot. To collect data for this experiment the robot platform was kept stationary while it whisked into a pole at varying radial distances to contact, and whisk speed. **(B)** The CrunchBot mobile whiskered robot.

The SCRATCHbot robot platform (Figure 2A) consists of a head-mounted whisker array, a mobile body housing computing means, motors and power supply, and an articulated neck allowing free movement of the head independent from the body. For this experiment we focus only on the head. Six independent columns of three whiskers, arranged in two arrays of nine whiskers either side of the head, are independently driven by a DC motor and gearbox. Whiskers in a column are mechanically coupled, but columns themselves are capable of independent rotational (anterior–posterior) whisk-like movement. Movements and data collection are coordinated by independent micro-controllers. A central PC-104+ reconfigurable computing platform, including a closely coupled array of FPGAs and a single board computer, handled all sensor and motor coordination.

#### 2.1.3. CrunchBot robot platform

CrunchBot (Figure 2B) consists of an iRobot Create base (irobot.com) with an extended cargo bay to accommodate a netbook PC. This netbook is used for autonomous control of the robot, running Ubuntu 10.10 on a single-core Intel Atom processor. The netbook hosts a Player server (playerstage.sourceforge.net) providing high-level, networked API interfacing to the Create's serial port commands. Rapid prototyped ball joint mountings fixed to an adjustable metal bar individually hold six static artificial whiskers. The whiskers are positioned at angles to fan out across the width of the robot while covering any blind spots. Radial distance estimation and basic motor control can run in real time on the netbook, reading the raw data from the circular buffer.

### 2.2. Artificial whiskers

**Figure 3:**
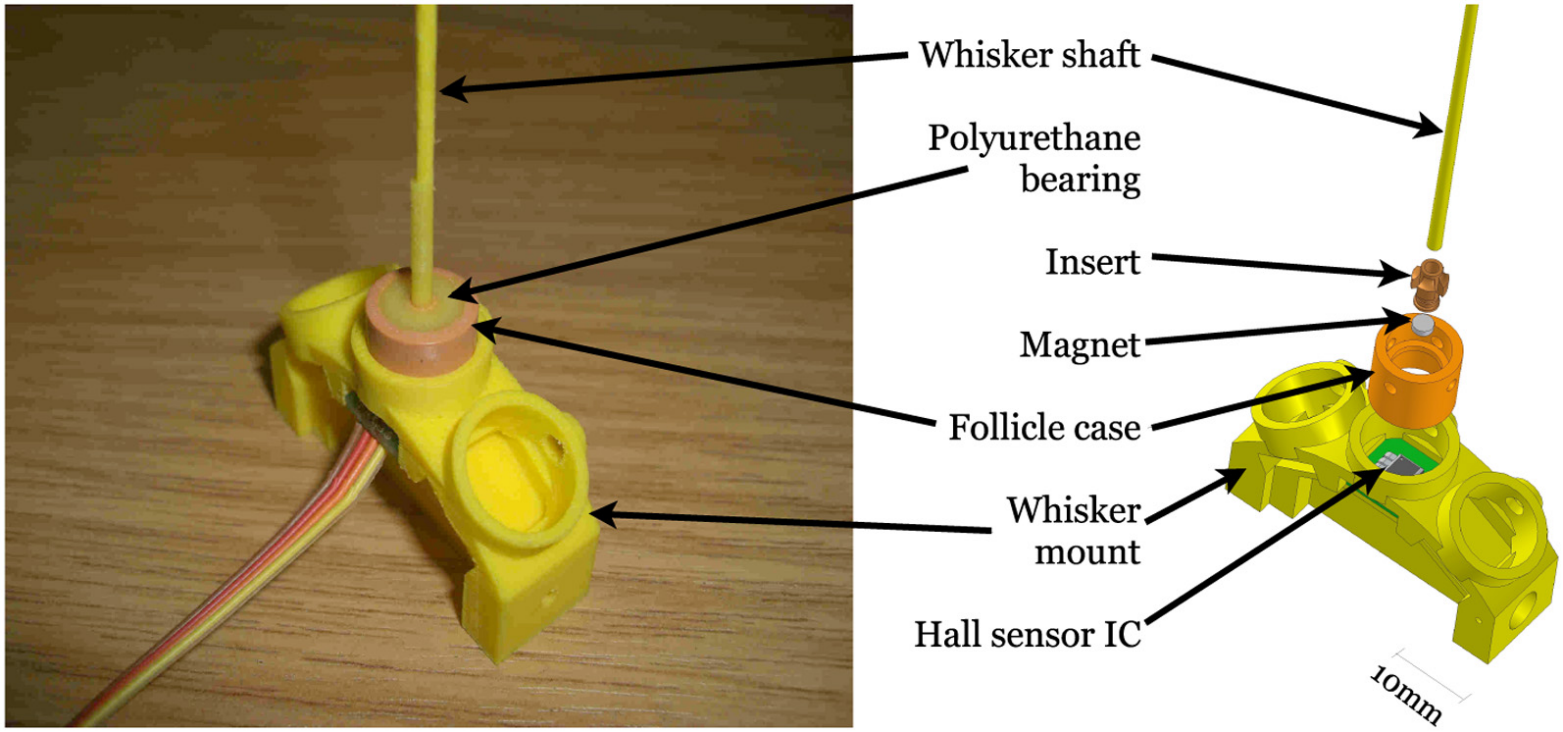
**Diagram of whisker follicle sensor construction.** CrunchBot whisker follicles differ slightly in shape, but operate in exactly the same way.

Three different materials were used in the fabrication of whiskers for the three robot platforms. Each whisker is made on an Envisiontec Perfactory rapid prototyping machine (envisiontec.de). The XY positioning robot whisker was made from flexible Acrylonitrile butadiene styrene (ABS) plastic (*E* ≈ 1.63 GPa, ζ ≈ 0.07), 185 mm long, 2 mm diameter at the base, 0.5 mm at the tip. SCRATCHbot whiskers are identical in shape and size to those on the XY positioning robot, but are made from the fiberglass material (*E* ≈ 25 GPa, ζ ≈ 0.5). CrunchBot whiskers were made from Nanocure RC25 (*E* ≈ 4.89 GPa, ζ ≈ 0.2) and were smaller in size, 160 mm in length, 1.45 mm diameter at the base tapering linearly to 0.3 mm at the tip. All whiskers are straight, but may curve slightly due to gravity perpendicular to the plane of movement, and are linearly tapered. Each whisker was mounted at the base into a short, polyurethane rubber (Poly 74-20 RTV from Polytec, synergyrm.co.il) filled, inflexible tube called a follicle case (see Figure 3).

A magnet was bonded to the base of the whisker shaft in such a way that when the follicle case/whisker shaft assembly was located into the whisker mount (see Figure 3), the magnet was positioned directly above a tri-axis Hall effect sensor integrated circuit (IC, Melexis MLX90333 www.melexis.com). Hall effect sensors measure the change in voltage across a conductor in response to changes in the strength of a nearby magnetic field. The tri-axis Hall effect sensor used here can measure the voltage changes in three orthogonal axes, i.e., *x* and *y* across the plane of the sensor, and *z* upwards toward the whisker. As forces are applied to the whisker shaft, the moment experienced at the base will rotate the magnet around a pivot point, nominally in the center of the polyurethane bearing. The sensor output voltage provides information about the magnitude of whisker deflection whether the whisker is moving or not, therefore the information is useful for static as well as dynamic classification approaches. When the whisker is deflected the movement of the magnet is proportional to whisker bending.

### 2.3. Data collection

#### 2.3.1. XY positioning robot data collection

Deflections of the whisker were transmitted through the Hall effect sensors to a LabJack UE9 USB data acquisition card (labjack.com) at a rate of 1 kHz for each of the *x* and *y* directions. Each trial lasted 4 s. This data was sent to a computer through the BRAHMS middleware (brahms.sourceforge.net) for analysis in MATLAB.

A MI control policy (observed in rats and discussed in Section 1.1) was implemented. In contrast to passive deflections, this policy keeps the amplitude and duration of whisker deflection within a limited range, and also keeps whisker ringing after contact to a minimum. An additional benefit is that the forces acting on the whisker are much smaller, meaning whisker breakage is less likely, even in high speed collisions.

MI was implemented by instructing the robot to move an object (here a narrow, rigid, cylindrical bar) into the whisker at a given speed until a deflection magnitude threshold (0.05 V) is crossed, at which point the robot retracts the object as fast as possible (720 mm/s). Temporal latency for the loop is ≈300 ms from initial contact due to the controller duty cycle.

Preliminary investigations showed that contacts could be made over a radial distance range of 80–180 mm without saturating the Hall effect sensor, or the bar slipping past the whisker tip before a retraction. Object speed ranged from 36–216 mm/s. Contacts were sampled at radial distance intervals of 1 mm, and speed intervals of ≈7 mm/s over the previously described ranges, respectively. In total 101 radial distances and 26 speeds were sampled, giving 2626 different radial distance and speed combinations. Contact combinations were randomly interleaved during data collection to limit any order effects, such as changing whisker properties across trials. For each contact combination, the whisker was deflected by the robot in both a clockwise and anticlockwise directions (−ve and +ve in *x*, see Figure 1), ensuring that the whisker did not undergo plastic deformations. The experiment was performed twice (two runs of clockwise and anticlockwise, generating four separate sets in total) to generate sufficient data for training the classifier. Data from each trial was stored separately. Deflections from the clockwise robot movement trials (−ve in *x*) were multiplied by −1, so data from all trials were directly comparable. Trials were ordered into arrays by speed and radial distance to contact. Each trial was aligned to peak deflection, and cut down to only 325 ms either side of the peak deflection.

#### 2.3.2. SCRATCHbot data collection

A single column of whiskers from a SCRATCHbot head was used for this experiment. The upper and lower whiskers were removed, and the dorsal–ventral axis of the whisker was set to 90° (horizontal). A vertical aluminium bar (13 mm cross-sectional diameter) was positioned at three different radial distances (70, 100, and 130 mm) at an azimuthal angle of 135° (180° = dead-ahead). The whisker was driven around the azimuthal axis (anterior–posterior) using a sinusoidal whisking pattern, with a retraction-protraction range of 60° (from 90° to 150°). The frequency of whisking was set at 2, 4, and 6 Hz, giving nine conditions in total. Eight contacts were made in each condition, four were used for training the classifier and four for testing (36 contacts for both training and testing). Data was streamed to the onboard computer through BRAHMS, and stored for later analysis. The whisker drive controller received no sensory feedback from the whisker sensor itself, only using the absolute measurement of theta to close a PID controller.

#### 2.3.3. CrunchBot data collection

Data from the six whiskers was collected using an FPGA configured as a bridge to a USB 2.0 interface. Up to 28 whiskers can be connected to this FPGA bridge at one time. Using the vendor provided software driver and API (Cesys GmbH http://www.cesys.com/en/home.html), a user can request the data from all whiskers at minimum intervals of 500 μs (a sample rate of 2 kHz).

A “body whisk” behavior was included in the robot program to ensure consistent contact forces and speed. As the whiskers were not actuated the whole robot must rotate in a systematic way to simulate the whisking behavior of rats. Upon initial contact with an object the robot first reverses away a short distance before rotating at 15° per second toward the object for 1 s, then rotating at 15° per second away from the object for 1 s. This allows this whiskers to move over the surface of the contact object, collecting data about the radial distance (or in other experiments the orientation and texture of the surface). After the whisk the robot reverses again to clear the object, then rotates in a random direction and moves forward again. The whisker sweep during the contact phase is similar to a sinusoidal whisk.

For the verification of radial distance estimation a square cornered object was used. The robot was set in motion on a trajectory that would ensure the corner of the object would make contact with a particular whisker at a specific radial distance. The robot would then perform the body whisk movement, and the data would be stored. A dataset was collected for each whisker, consisting of five contacts at each of six points along the whisker (10 mm intervals over a 50 mm range) from the tip of the whisker. Though the whisker is 160 mm long, only 140 mm is external to the “follicle.”

### 2.4. Feature-based radial distance estimation with uncertain contact speeds

To successfully implement a feature-based classifier, appropriate features must first be found and extracted. Inspection of the whisker data showed that Hall effect sensor output voltage at peak deflection (proportional to bending moment *M*) could be used as a feature for radial distance discrimination at a given speed.

Feature *f*_1_ can be defined as

(1) $$f_{1}=\max_{t}M(t),$$

where *M*(*t*) is the deflection magnitude varying with time, measured by the Hall effect sensor in volts. Note that *t*(*f*_1_) is the time at max_*t*_ *M*(*t*).

**Figure 4:**
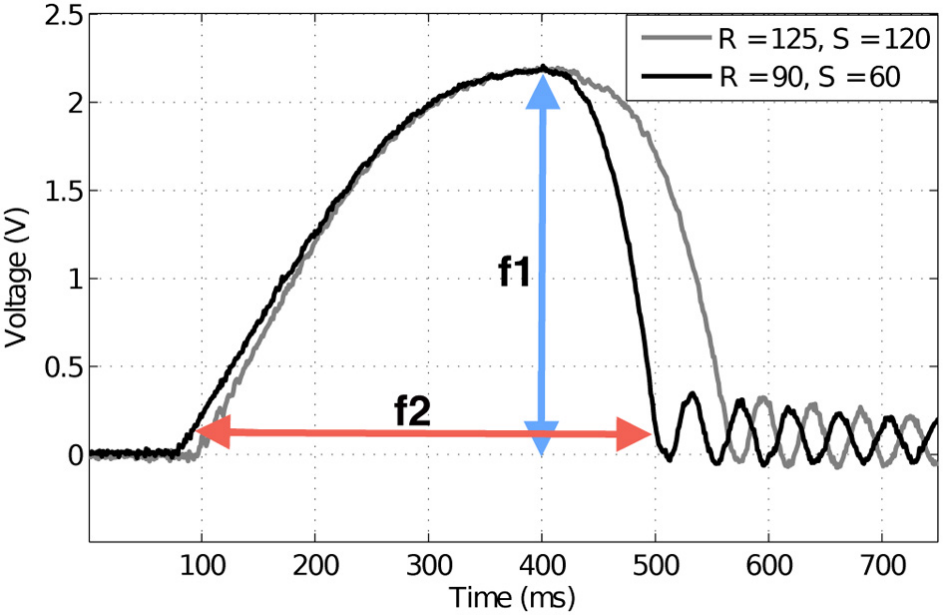
**Example deflection signals from the artificial whisker.** Magnitude of deflection, or force, has been used previously as a discriminator of radial distance to contact. Here the two traces are at different radial distances (measured in mm), but create the same magnitude of deflection. Speed measured in mm/s. Colored arrows indicate how the extracted features for classification are measured. Peak deflection magnitude *f*_1_ (blue arrow) and contact duration *f*_2_ (red arrow) are used to discriminate radial distance to contact and contact speed, respectively.

Similarly, contact speed could be discriminated using deflection duration. Deflection duration was taken as the width of the deflection peak (prominent initial deflection in each trace of Figure 4). Deflection duration was measured using a threshold crossing on the sensor output. When Hall effect sensor output exceeded γ = 0.05 V a timer was initiated (*t*_1_), and when Hall output subsequently fell below this threshold the timer was stopped (*t*_2_). Feature *f*_2_ (measured in ms) can thus be defined as,

(2) $$t_{1}=\min\{t:M(t)\geq \gamma \},$$

(3) $$t_{2}=\min\{t:M(t)\leq \gamma ,t_{2}>t_{1}\},$$

(4) $$f_{2}=t_{2}-t_{1},$$

where γ is the threshold. Colored arrows in Figure 4 give examples of these measurements.

A model was generated of the relationship between each pair of features and the corresponding contact properties with polynomial regression (using polyfitn in MATLAB, bit.ly/polyfitN). Using linear least squares a model is generated that can be used to classify new data. Three arguments are required for the model, an array of independent variable values, an array of dependent variable values, and a model specification, namely the degree of the polynomial. A fifth degree polynomial was chosen as preliminary studies showed it provided good results. The independent variables in this instance were features *f*_1_ and *f*_2_. To find both radial distance and speed, two models were developed, with dependent variables of radial distance and speed, respectively.

The same polynomial regression operation was repeated on data from SCRATCHbot, though fewer summary statistics were generated as the dataset was smaller than that generated on the XY positioning robot.

On CrunchBot a linear regression was used. As robot motion is controlled, whisker contact speed variability is low between trials and a linear regression is sufficient for classification in this instance. To find an estimate of radial distance *r*,

(5) $$r=a_{1}f_{1}+a_{0},$$

was fitted to the data with a linear-in-the-parameters regression on the line, giving a least-squares fit for (*a*_0_, *a*_1_) for each whisker. Due to the small dataset size a “leave one out” protocol was used for classifier testing of CrunchBot. Four out of five contacts at each radial distance was used to train the classifier, with the remaining contact used for testing. This process was repeated using a different test contact each time. For each robot a mean absolute error statistic is given, which is more informative than mean error alone.

## 3. Results

### 3.1. XY positioning robot

**Figure 5:**
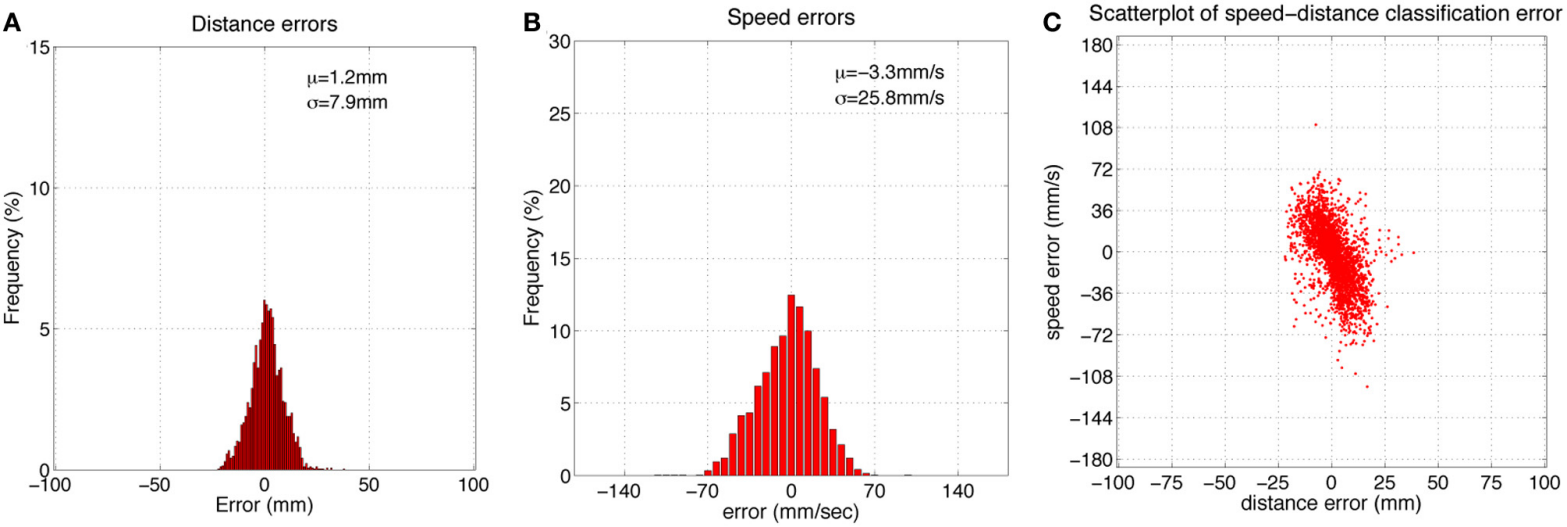
**(A,B)** Histograms of radial distance and speed classification errors using the feature-based classifier. **(C)** Scatterplot of these errors for each point in the dataset. μ = mean, σ = standard deviation, sample size = 2626.

**Figure 6:**
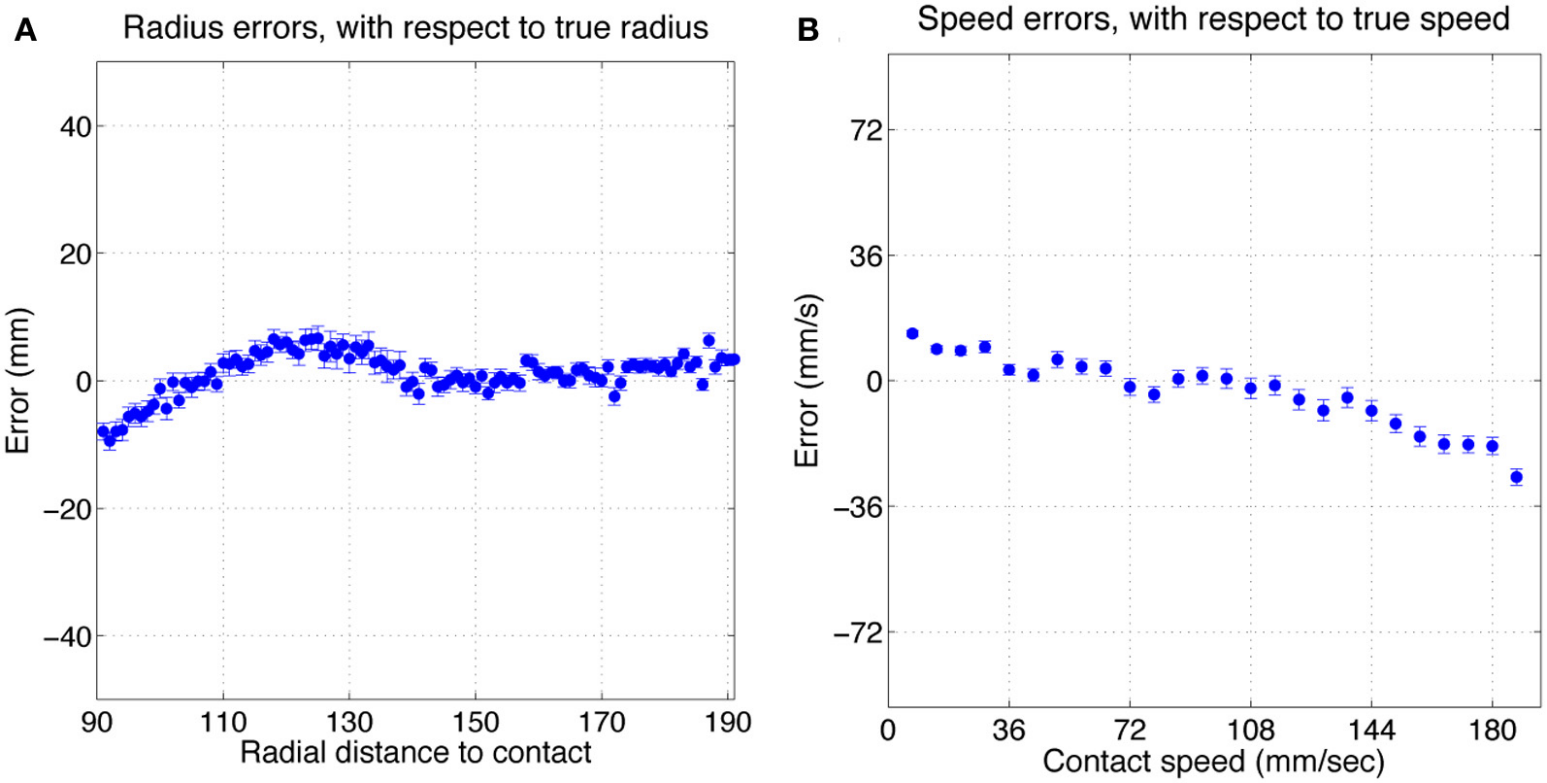
**Mean classification error for radial distance error, with respect to true radial distance (A), and for contact speed, with respect to true contact speed (B).** Errorbars show standard error.

Figure 5 shows histograms of classification errors for both radial distance **(A)** and speed **(B)**, and a scatterplot of the errors for each sample in the test set. Mean μ and standard deviation σ for radial distance and speed estimation errors was 1.2, 7.9 mm and 3.3, 25.8 mm/s, respectively. The mean absolute error was 6.2 mm and 20.4 mm/s for radial distance and speed, respectively. Figure 6 shows mean classification error for radial distance error, with respect to true radial distance **(A)**, and for contact speed, with respect to true contact speed **(B)**.

**Figure 7:**
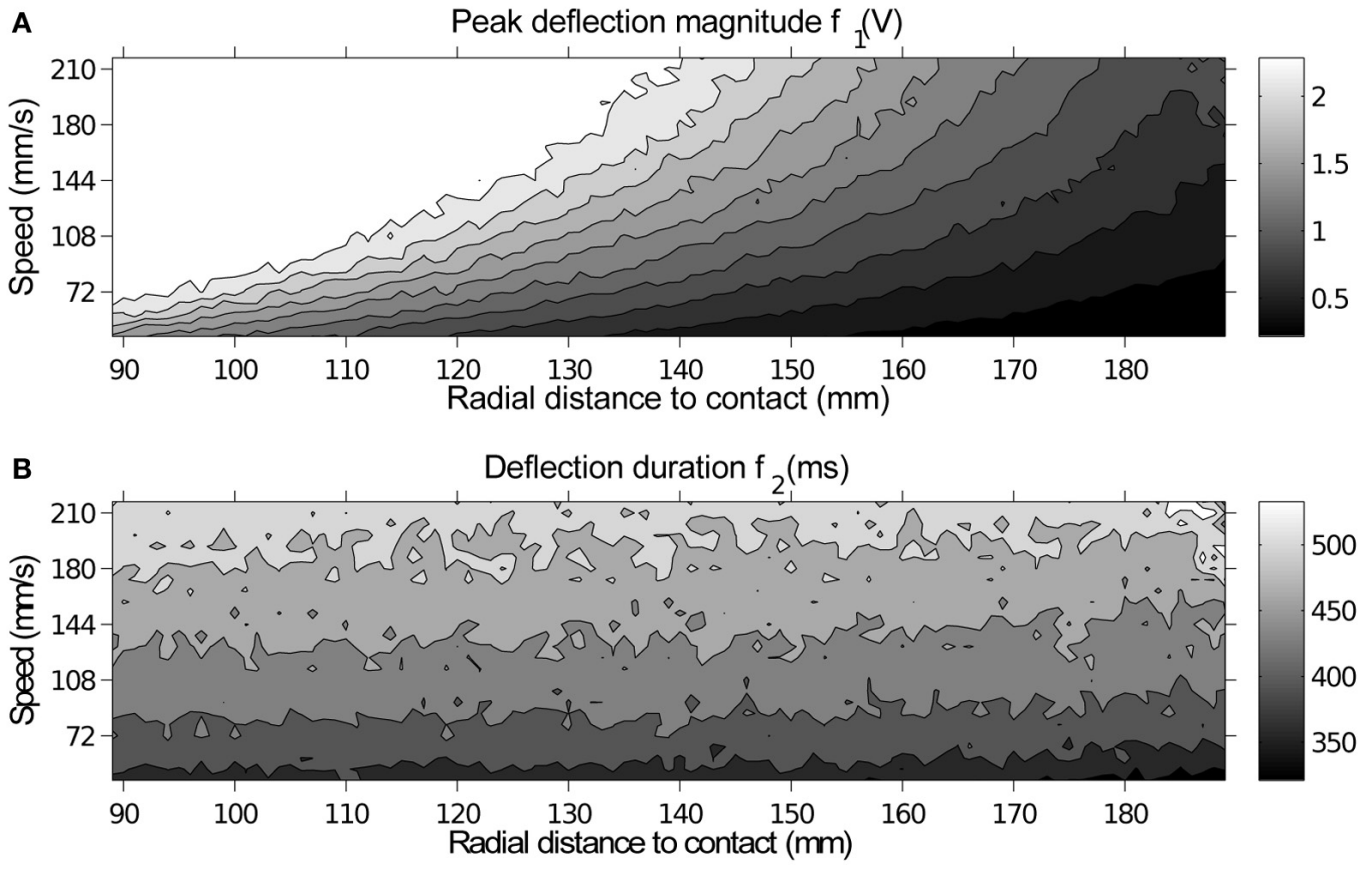
**A contour plot of peak deflection magnitude and duration for each contact showing how each feature varies with respect to contact parameters.** Each point in the image corresponds to a location in the speed-radial distance space, which is equivalent in both plots. **(A)** Peak deflection magnitude *f*_1_, brightness indicates higher deflection magnitude, measured in volts. All 10 contours are evenly spaced across the voltage range. **(B)** Deflection duration *f*_2_, brightness indicates greater duration (measured in ms). All 6 contours are evenly spaced across the duration range.

Figure 7 shows a contour plot of the extracted features *f*_1_ and *f*_2_. While deflection magnitude is proportional to radial distance to contact for a given speed (Figure 7A), the precise degree of deflection is ambiguous without a separate measure of contact speed. On data generated on the XY positioning robot the duration of contact can provide this additional measure. This figure is examined further in Section 4.

### 3.2. SCRATCHbot

**Figure 8:**
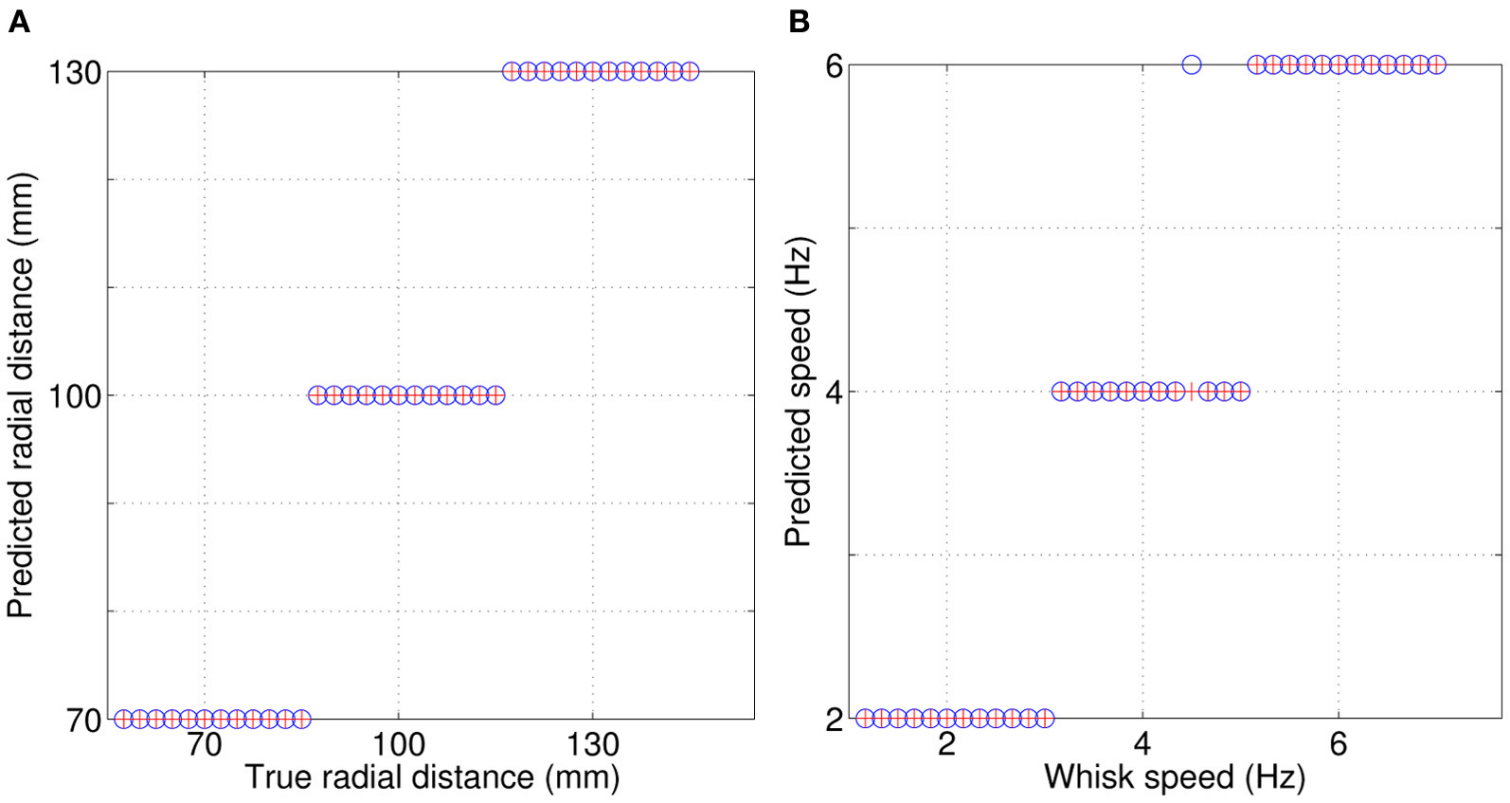
**Simultaneous classification of radial distance to contact (A), and whisk speed (B) on SCRATCHbot.** Red crosses = true values, blue circles = classifications. Only one contact is miss-classified in the 36 contact test-dataset.

**Figure 9:**
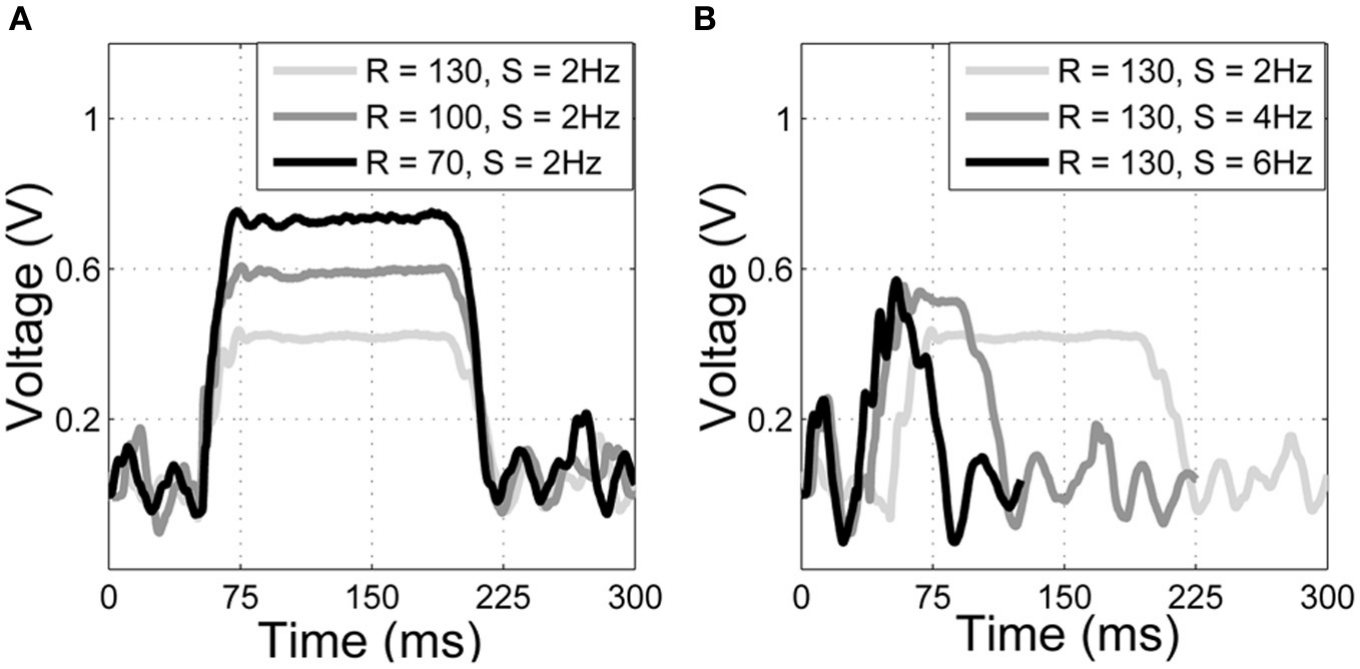
**Raw data from SCRATCHbot.** Properties of the deflections match closely to those from the XY positioning robot (compare with Figure 4). **(A)** three deflections at different radial distances (R, in mm), but the same speed (S, in Hz). Peak deflection height varies predictably with radial distance. **(B)** three deflections at the same radial distance but at different speeds. Contact duration varies predictably with speed. Contact latencies are for clarity of presentation.

Results from SCRATCHbot show that the features and classifier developed on the XY positioning robot also apply to data collected from a whisking robot. As Figure 8 shows, classification performance is almost perfect, with only one mis-classification of speed in the 36 contact test-dataset. Figure 9 demonstrates that a key difference between data from SCRATCHbot and the XY positioning robot is the way whisker speed affects contact duration. Contact duration on the XY positioning robot increases as object speed increases, as object retraction is controlled by a feedback loop of a fixed duration. The faster the object moves, the further the whisker is deflected before a retraction is initiated. This increases contact duration in proportion to an increase in speed. Since SCRATCHbot is performing active whisking onto a static object, increased whisk speed results in a shorter contact duration. However, though the direction of the relationship is reversed, whisk speed still predictably affects contact duration. As in the XY positioning robot data, whisking at the same speed but different radial distances affects peak deflection magnitude (as can be seen in Figure 9B). Taking contact duration into account with a feature-based classifier allows accurate radial distance estimation at different whisk speeds.

### 3.3. CrunchBot

**Figure 10:**
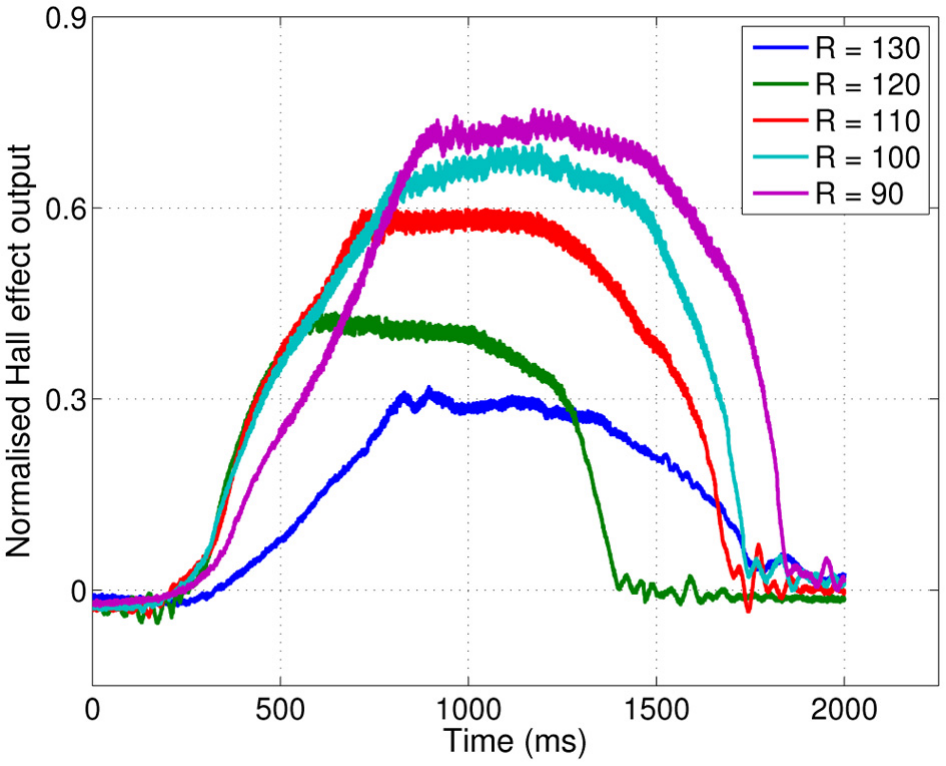
**Radial distance to contact affects deflection magnitude on the mobile CrunchBot robot.** Five deflections at different radial distances (R, in mm), but the same speed. Peak deflection height varies predictably with radial distance.

**Figure 11:**
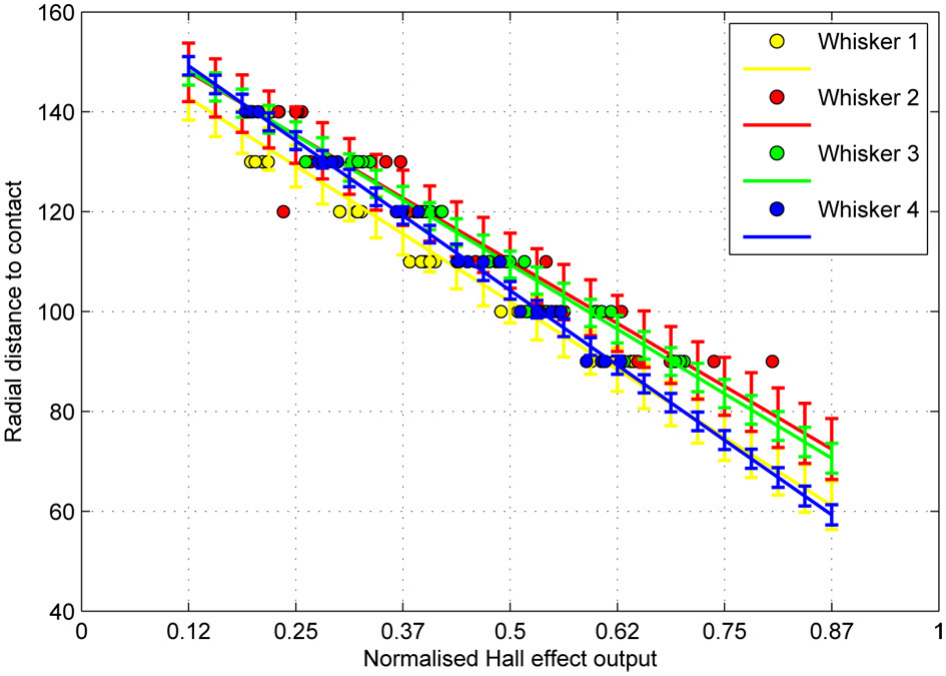
**Radial distance to contact for a given magnitude of whisker deflection (dots), and estimates of the standard deviation of the error in predicting future observations (errorbars) for each whisker.** Sample size = 30 contacts per whisker, 120 in total. Radius measured in mm.

Typical whisker deflections from CrunchBot are shown in Figure 10. Peak deflection magnitude for each contact is shown in Figure 11. Mean absolute error for radial distance estimation is shown in Table below.

**Table:** 

	**Whisker 1 (mm)**	**Whisker 2 (mm)**	**Whisker 3 (mm)**	**Whisker 4 (mm)**	**Combined (mm)**
μ abs. Err	4.11	1.89	1.28	3.30	2.65

Mean absolute error is very low, typically less than 5 mm over the 50 mm range tested. For some whiskers classification error is even lower, below 2 mm. These results compare favorably with results from controlled conditions on the XY positioning robot (Section 3.1) where speed was variable. This indicates that the noise in the odometry is low enough to ensure a consistent contact force and speed on this mobile robot.

## 4. Discussion

Deflection magnitude is proportional to radial distance to contact, a relationship that is preserved across robot platforms, regardless of whisker material or actuation method. Whisker-object contact speed also affects deflection magnitude in a predictable manner on both the XY positioning robot and whisking SCRATCHbot. Controlling the whisker movement allows a very simple linear regression-based radial distance estimation method to be successfully implemented on CrunchBot, a mobile robot.

### 4.1. Comparison and synthesis across robot platforms

It may have been assumed that the relationship between whisker deflection, for a given radial distance to contact, and contact speed would be linear. However, though the relationship may be linear for a certain radial distance to contact, that linear relationship does not hold for all contact locations along the whisker. This can be seen on data from the XY positioning robot in Figure 7.

Inspection of Figure 5C reveals that classification errors on XY positioning robot data are not completely random. There is an interaction between the parameters, which can be seen as a skewing in alignment of the errors: positive errors in radial distance estimation occur more often with negative errors in speed estimation, and vice versa. A more detailed look at these effects can be seen in Figure 6. This figure shows how classification errors vary across each parameter range. The systematic trends reflect the fact both speed and radial distance are classified simultaneously. Both contact speed and radial distance estimation is best (error is lowest) in the middle of each range. Large radial distances are over estimated (negative errors on the left side of Figure 6A) and small radial distances are underestimated (positive errors on the right side of Figure 6A). The opposite effect is seen for contact speed (Figure 6B), where large contact speeds are underestimated and low contact speeds are overestimated.

These effects can be explained by looking at how the extracted features change across the parameter space in Figure 7. A positive classification error of speed i.e., a jump from a dark to light area in Figure 7B, would result in a corresponding negative classification error of radial distance i.e., a jump from a dark to light area in Figure 7A. More transparently an increase in contact speed results in an increase in contact duration (*f*_2_), while an increase in radial distance to contact results in a reduction in deflection magnitude. Misclassifications as the deflection “grows” in both height and duration would result in an over-estimation of speed and an underestimation of radial distance and vice versa. A prediction of this work for biological whisker systems is that rats would overestimate the radial distance to contact when contact speed is lower that expected, for example, as an object moves away from the rat.

All three robotic platforms presented here use different whisker materials and control strategies. These differences affect the temporal pattern of whisker deflections, which can be seen in Figures 4, 9, and 10. Whisker mechanical properties affect the initial rate of deflection change, and contact induced oscillations. Stiff fiberglass SCRATCHbot whiskers (Figure 9) result in a sharp initial increase in deflection and larger oscillations in between contacts. On the XY positioning robot and CrunchBot (Figures 4 and 10, respectively) robot movement speed changes throughout the contact, slowing down as peak deflection is approached, resulting in differences in gross deflection shape. While previously work has shown that whisker movement affects texture discrimination (Evans et al., 2009; Lepora et al., 2012a; Sullivan et al., 2012), our results show that such changes do not affect the key features extracted for radial distance estimation with the feature-based method presented here. Successful classification of radial distance on a particular robot platform does require the classifier to be trained on data from that robot, but the underlying principles are invariant for whisker material and robot movement. Specifically, that radial distance to contact affects the magnitude of peak deflection, and this is modulated predictably by contact speed.

### 4.2. Relation to other studies of radial distance estimation

These are the first published results of whisker-based contact speed estimation. As rats carefully control whisker motion, and as consequence contact speed, it may not be immediately apparent why this discrimination is important. Objects in the environment sometimes move and, for example, when a shrew is hunting crickets it needs to determine both the location and movement of that prey animal to execute an accurate fatal attack (Anjum et al., 2006). Another consideration is that rats have no spindles in their whisking muscles, and therefore do not have accurate proprioception of their whiskers (Diamond et al., 2008; Mameli et al., 2010). Since accurate radial distance estimation is dependent on well characterized contact speed, a signal-based method, such as the contact duration feature approach presented here, is of potentially great importance. From a robotics perspective this kind of tactile movement tracking may be useful for other tasks, such as in tactile manipulation.

It is difficult to determine how much better or worse the feature-based approach presented here is over previous whisker-based radial distance estimation methods. These are the first results where contact speed is both variable and unknown. On data collected on the XY positioning robot we report a mean absolute discrimination error of 6.17 mm with a 185 mm whisker. This is a normalized accuracy of 3.4% of whisker length. In a real-time application on board a mobile robot we report an average mean absolute discrimination error of 2.65 mm with a 160 mm whisker. This is a normalized accuracy of 1.65% of whisker length.

With fixed contact speed and a static beam equation-based method (Solomon and Hartmann, 2010) report contact localization accuracy between 0.3 and 0.88 mm on different surfaces with a 50 mm whisker. This is a normalized accuracy of ≈1% of whisker length. This approach is more accurate than the results presented in the present paper, but reported under very different conditions (contact speed was very carefully controlled, slow, and not variable across trials). Further research is required to determine whether a similar approach would be successful in a more applied mobile robot setting, or under conditions of variable contact speed.

Rats have demonstrated radial distance estimation up to an accuracy of 2.5 mm (Krupa et al., 2001), which, with a 50–60 mm whisker, is a normalized accuracy of ≈4–5% of whisker length. These findings indicate that the feature-based approach presented here compares favorably with the performance of rats, even in the strict conditions of single whiskers making single object contacts and variable contact speed.

The results compare less favorably with typical range finding methods in robotics, such as laser range finders, or a Microsoft Kinect camera, which are both capable of sub-millimeter accuracy over short ranges (Khoshelham, 2011). Therefore the key contribution of whisker sensors for robotics is unlikely to be contact localization for its own sake, but in a wide range of other applied settings. Firstly, whiskers are useful in environments where other localization methods are impaired, for example, in smoky and dusty environments or underwater. Secondly, whiskers are small, low powered and can be manufactured cheaply, making them ideal for implementation as arrays on mobile robots. Finally, accurate characterization of contact properties such as localization and speed are essential for subsequent surface discriminations, as previous results have shown that whisker-based texture discrimination is improved when contact location and whisker movement are taken into account (Fend, 2005; Evans et al., 2009; Fox et al., 2009). A feature-based approach, as demonstrated here, could in principle provide a texture classifier with the necessary contact localization and speed information for improved discrimination. The integration of multiple texture reports over time into a local map of an object would also be dependent on accurate contact localization. This would be an extension of the tactile SLAM work published previously (Fox et al., 2012), and an area we hope to pursue in the future.

Hall effect sensors have some advantages over other sensing methods. Hall effect sensors are robust to damage, especially when housed in a rubber filled follicle, which is an important consideration when measuring whisker deflections as they are constantly striking objects in the environment. Hall effect sensors are also relatively inexpensive, and can be made quite small which makes them ideal for application to large arrays of whiskers. It has been proposed that rats determine radial distance to contact by encoding the bending of whiskers through moments at the base (Szwed et al., 2006). The Hall effect sensor is not a direct model of the rat follicle sinus, and does not report pure moments or forces at the whisker base but a combination of these properties along with whisker rotation angle about a pivot. Using a completely hard follicle rubber would remove the angular component of the deflection, but the sensor would no longer be able to measure bending. A whisker sensor could feasibly be designed that more closely models the physical structure of the rat follicle sinus, but it would remain an approximation. The artificial whiskers presented here capture the important aspects of contact induced deflections at both high and low frequencies, which is sufficient for understanding the abstracted principles of whisker sensing.

### 4.3. Implications for understanding biological whisker systems

Exploring active whisker movement in artificial systems highlights that active whisker control may be similar to aspects of eye movement control in active vision (Aloimonos et al., 1988). The field of active vision explores how the eyes may be moved to efficiently search an environment. The difference between active vision and active touch is in the scale of the movements with respect to the environment. In active vision the sensors can be moved to search a whole environment, for tasks such as scene identification of mapping (Davison and Murray, 2002). Active whisker touch can only be used over a very local region of the environment, therefore active whisker control may be thought of as analogous to micro-saccades for gaze stabilization (Collewijn and Kowler, 2008) or pupil diameter and lens focus, for luminance and depth of field control in the eye (Koss and Wang, 1972; Takehiko and Haruo, 1991). In vision optic flow and retinal slip can be used as an error signal for corrective eye movements for smooth pursuit (De Brouwer et al., 2001). Similarly, a measure of contact duration may be used by the rat as an error signal to correct whisker movements.

## 5. Conclusion

In this paper we have shown that a similar complementary approach can be successfully pursued in robotics. Certain experiments are much easier to perform on robots with fewer degrees of freedom, such as the XY positioning robot. The results from these experiments can save a great deal of time when implementing classifiers onboard mobile robots such as CrunchBot, or robots with high degrees of freedom such as SCRATCHbot. Mobile robot experiments can then generate predictions for biological systems, or drive further XY positioning robot research. This approach, and indeed these robots, could be used to answer a broad array of questions about active touch in the future.

We have shown that in each of the robots presented here, regardless of whisker material or actuation method, the radial distance to contact can be determined from peak deflection magnitude. In addition the speed of contact also predictably affects the amplitude of whisker deflection in each of these robots. By taking the speed of contact into account, radial distance estimation can be accurately performed in a range of settings. We predict that if whiskered mammals are using deflection amplitude (or degree of bending) to determine the radial distance to contact, the contact induced signal will change if the animal whisks at a different speed or force, and that this must be taken into account for accurate discriminations.

### Conflict of interest statement

The authors declare that the research was conducted in the absence of any commercial or financial relationships that could be construed as a potential conflict of interest.
